# Death following bilateral complete Achilles tendon rupture in a patient on fluoroquinolone therapy: a case report

**DOI:** 10.1186/1752-1947-3-1

**Published:** 2009-01-06

**Authors:** Andrew W Gottschalk, John W Bachman

**Affiliations:** 1 Mayo Clinic, Department of Family Medicine, 1st Street SW, Rochester, MN 55905, USA

## Abstract

**Introduction:**

Risk of tendon rupture, especially of the Achilles tendon, is one of the many potential side-effects of fluoroquinolone therapy. Achilles tendon rupture may be painful, debilitating or, as seen in our patient, devastating. While fluoroquinolone-induced tendon rupture typically accompanies other comorbidities (for example renal impairment) or concurrent steroid therapy, our case represents a medical 'first' in that there were no such comorbidities and no steroid therapy. Furthermore, our case is remarkable in that tendon rupture was bilateral, complete, and resulted in a devastating outcome.

**Case presentation:**

A healthy 91-year-old Caucasian man was placed on fluoroquinolone (levofloxacin) therapy for a presumed bacterial pneumonitis. Subsequently, he developed bilateral heel pain, edema, and ecchymoses leading to a diagnosis of bilateral complete Achilles tendon rupture. This drug's side-effect was directly responsible for his subsequent physical and psychologic decline and unfortunate death.

**Conclusion:**

Fluoroquinolones are a powerful and potent tool in the fight against bacterial infection. As a class, they are employed by primary care physicians as well as by subspecialty physicians in all areas of medical practice. However, as this case illustrates, the use of these drugs is not without risk. Attention must be paid to potential side-effects when prescribing any medication, and close follow-up with patients is a medical necessity to evaluate for these adverse reactions, especially with fluoroquinolones.

## Introduction

Fluoroquinolones treat a broad spectrum of both Gram-positive and Gram-negative organisms, have high enteral bioavailability, and are renally excreted. Fluoroquinolones are effective in respiratory, urinary, and gastrointestinal tract infections. Furthermore, their rapid accumulation in bone and cartilage makes them a convenient and effective therapy in the field of orthopedics [1]. For all of these reasons, fluoroquinolones are an attractive antimicrobial choice of treatment among healthcare providers in most fields of medical practice.

However, the treatment is not without its cost. Fluoroquinolone accumulation in developing joints has raised concerns based on studies of laboratory animals on the potential for cartilage erosion and subsequent arthropathy. They are therefore contraindicated in obstetric patients, breastfeeding mothers, and in pediatric patients younger than 18 years old. Side-effects from fluoroquinolone use include arrhythmias, hypoglycemia, pancytopenias, hepatitis, polyneuropathies, and acute renal failure [2,3]. Another side-effect of concern is tendinopathy, with ciprofloxacin, norfloxacin, ofloxacin, and levofloxacin having associations that have been reported in the literature [1,4-7]. Tendinopathy subsequent to fluoroquinolone treatment, typically classified as tendonitis (inflammatory) or tendinosis (microtears), has an incidence of 2.4 per 10 000 patients. Partial Achilles tendon rupture is rarer still, at 1.2 per 10 000 [8]. However, neither complete tendon rupture nor bilateral tendon rupture occur often enough to have a calculated incidence in fluoroquinolone-treated patients; rarer still are the two conditions occurring together.

## Case presentation

**Figure 1:**
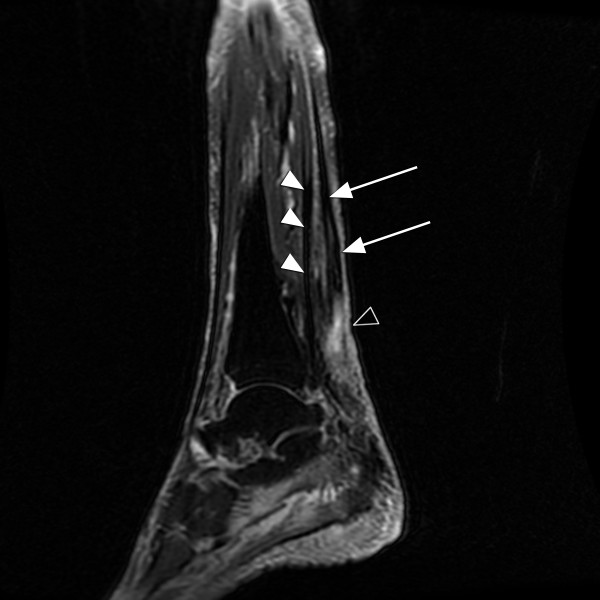
**T2-weighted image of the patient's left heel**. MRI machines measure proton density; density is proportional to how dark a tissue appears on the scan. 'T1' and 'T2' are technical terms describing the time required for proton relaxation. T2 images make adipose tissue, water, and other fluids bright, thus these images are ideal for detecting tissue edema. On the image, note the intact flexor hallucis longus (filled arrowheads) pulled taught. The flexor hallucis longus is responsible for what little plantar flexion the patient had left. The arrows show the retracted and limp proximal end of the Achilles tendon, with the bright area of signal intensity (open arrowhead) representing inflammation and fluid migration between the severed ends.

**Figure 2:**
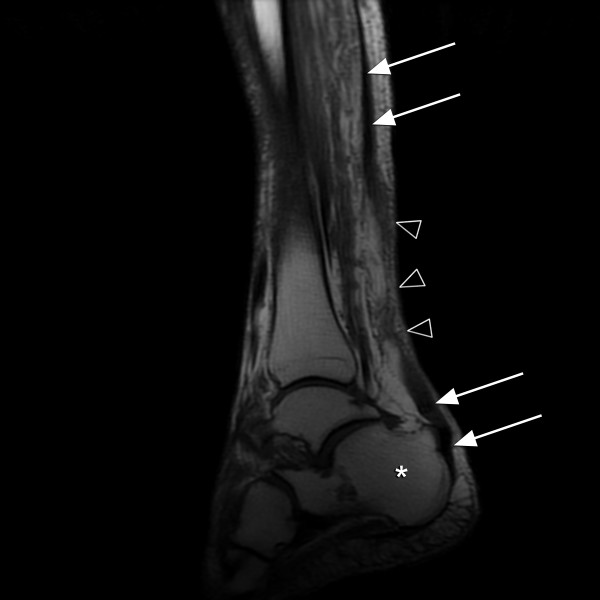
**T1-weighted image of the patient's right heel**. T1 images do not amplify the brightness of low-density tissue, and therefore do not risk obscuring pathologic findings in more dense tissues, such as tendon and bone. On the image above, arrows follow the complete rupture of the Achilles tendon. Note how the distal end lays 'floppy' over the superior calcaneus (asterisk). The open arrow-heads note intervening inflammation and soft-tissue edema.

A 91-year-old Caucasian male farmer presented at the Mayo Clinic, Rochester, Minnesota for evaluation of the acute complaint, "My feet aren't working." Specifically, he reported difficulty with balance leading to inability to walk, and extensive, painful bilateral ecchymoses of his heels (Figures 1 and 2). His distress and instability were alleviated only modestly by the use of a non-prescription cane. Barefoot ambulation was impossible. Barely functional ambulation was accomplished by wearing hard-soled shoes.

One month prior, the patient had been diagnosed with bronchitis at an outside clinic and was treated with a seven-day course of levofloxacin 500 mg by mouth, taken once a day (his estimated creatinine clearance was 32 mL/min using the Cockcroft-Gault equation). His bilateral heel pain developed insidiously over the first four days of fluoroquinolone treatment.

Even at 91, the patient had led an active lifestyle on his farm. He cooked all of his own meals, drove a tractor, and performed many other physical farm chores. On day seven of treatment, upon dismounting his tractor, he noticed sudden, severe pain in both of his heels and a compromised ability to ambulate independently.

The patient's medical history was otherwise notable for a mitral valve replacement with porcine xenograft 25 years prior, chronic hypertension, hyperlipidemia, degenerative spondylosis, depression and gastroesophageal reflux disease (GERD). He had no history of tendinopathy. The patient had never smoked, and had no exposure to second-hand smoke. He reported alcohol ingestion of two beers per month and caffeine consumption of three 8 oz cups of coffee per day.

In addition to the levofloxacin, the patient was on the following medications, with no recent changes: metoprolol ER 100 mg/d, isosorbide mononitrate ER 60 mg/d, hydrochlorothiazide/triamterene 25 mg/37.5 mg/d, spironolactone/hydrochlorothiazide 25 mg/25 mg/d, quinapril 20 mg/d, celecoxib 200 mg/d, glucosamine 1500 mg/d, sertraline 100 mg/d, omeprazole 20 mg/d, and acetaminophen 1000 mg every six hours as needed for arthritis-related pain. He had no known food or medication allergies.

On presentation to the hospital, the patient was afebrile and all vital signs were stable. Both heels were mildly edematous with overlying ecchymoses and were tender to palpation bilaterally. The patient had a palpable gap and mass-like defect distally with palpation along the left Achilles tendon. There was a similar gap, although no palpable mass defect, on the right. Dorsalis pedis and posterior tibial pulses were 2+ bilaterally, and capillary refill was less than two seconds in the great toes bilaterally. Thompson's test (also known as Simmond's sign) was abnormal bilaterally, with no movement of either foot. The patient could barely raise himself up on his tiptoes. In both lower extremities, ankle plantar flexion strength was 3/5, great toe flexion to test the flexor hallucis longus was 5/5, and ankle dorsiflexion to test the extensor hallucis longus was 5/5. The patient maintained full active and passive knee range of motion bilaterally. He demonstrated antalgic gait with significant difficulty in the toe-off (propulsion) phases bilaterally.

Radiographs of the lower extremities noted soft-tissue edema of the ankles. There were no noted bony abnormalities.

The patient was initially admitted to the hospital for in-patient care and placed on non-weightbearing status. Magnetic resonance imaging (MRI) of the ankles noted complete rupture of both Achilles tendons approximately 6 cm proximal from insertion upon the calcaneus, with the two ends approximately 3 cm apart on the left and 2 cm apart on the right. The patient was fitted with gravity equinus casts with heel extensions to keep the feet in plantar flexion. He was discharged from the hospital one day after admission.

Five weeks after hospital admission, the patient's casts were removed and he was fitted with controlled ankle motion (CAM) boots that were in plantar flexion at 15 degrees with resultant full equinus. Nine weeks after hospitalization, the patient was instructed to stop wearing the CAM boots and began wearing his own tennis shoes with three-quarter inch heel lifts to maintain relative plantar flexion.

At his initial presentation to the outpatient care center on the day of admission, the patient's primary care physician noted, "History of depression and anxiety: He is not anxious and depression is currently not a problem. He looks much brighter." However, at the meeting with his orthopedic surgeon nine weeks after hospital discharge, both the patient and family members noted decreased energy levels and general lack of enthusiasm. There was concern that these symptoms were fueled by his immobility.

Ten weeks after diagnosis, the patient presented to the Emergency Department with a 22-pound weight loss over the prior two months as well as generalized lethargy. He was hospitalized for evaluation where he was hydrated and his antihypertensive regimen was modified (quinapril, spironolactone, and hydrochlorothiazide were discontinued). He was discharged with blood pressures well within the normal range and with close follow-up with his primary care physician.

The patient was readmitted for inpatient care the following day with hospital-acquired pneumonia. He subsequently developed kidney failure, sepsis, heart failure, and a myocardial infarction. After consultation with the patient and his family, care was withdrawn and comfort care measures were initiated until the patient passed away 11 weeks after the initial diagnosis of bilateral complete Achilles tendon rupture.

## Discussion

This is the first reported case of MRI-confirmed, bilateral complete Achilles tendon rupture in a patient on levofloxacin with no exposure to corticosteroids.

What makes this report so noteworthy, however, is not the severity of the side-effect, but the severity of the outcome. Although the adverse effects of these medications are numerous, never before in the literature has death been so evidently linked to the catalytic levofloxacin treatment. Perhaps this is because confounding pathologies and treatments, especially in the elderly, make that conclusion difficult to support. Although severe, however, this outcome should not be entirely unexpected. For example, hip fracture in the elderly has a post-incident mortality rate of one in four in the first year [9]. Over 6% of those deaths are in the first month alone, with outcomes dependent on both physical and mental health factors [9]. We would argue that tendon rupture can likewise be deadly. Physiologically, our patient could not perform the farm chores his body had become accustomed to and could no longer actively prepare his own meals. Psychologically, his mobility had allowed him to interact with his family, friends, and coworkers on the farm. Immobility led to social isolation which led to a recurrence of his depression. This, coupled with his rapid deconditioning, resulted in disaster.

## Conclusion

We argue that with Achilles tendon rupture, as with hip fracture in the elderly, 'the best offense is a good defense'. Although not used in our patient, steroid co-medication is a known risk factor for tendinopathy [5], and thus should be avoided when placing a patient on levofloxacin, or indeed on any fluoroquinolone. Patients currently on corticosteroid treatment should receive trials with other antibiotics before levofloxacin is considered. Doses should be adjusted accordingly in patients with decreased creatinine clearance. All patients should be educated as to possible side-effects of treatment. The development of tendonitis is an indication for discontinuing therapy, and informing patients of the possibility of tendon distress may prevent severe complications. Levofloxacin is an expensive, commonly used antibiotic. Although this fluoroquinolone is often appropriate therapy under certain circumstances, our case reminds us that levofloxacin therapy has associated risks, which in our patient catalyzed a downward spiral resulting in death.

## Consent

Written informed consent was obtained from the patient's family for publication of this case report and accompanying images. A copy of the written consent is available for review by the Editor-in-Chief of this journal.

## Competing interests

The authors declare that they have no competing interests.

## Authors' contributions

AG was the hospital physician admitting the patient, and is the main author of this manuscript. JB was the patient's primary care physician at the Mayo Clinic who collaborated on both patient care and this manuscript's conception. Both authors read and approved this final manuscript.
